# Motives for Vaccination Against COVID-19 Among the Ultra-orthodox Jewish Community in Israel

**DOI:** 10.1007/s10943-024-02018-3

**Published:** 2024-03-26

**Authors:** Miriam Schiff, Nitzan Sharon-Lavi

**Affiliations:** Paul Baerwald School of Social Work and Social Welfare, Hebrew University of Jerusalem, Jerusalem, Israel

**Keywords:** COVID-19, Vaccine uptake, Ultra-Orthodox Jews, Protection motivation theory (PMT), Gender differences

## Abstract

According to official data, the ultra-Orthodox group in Israel had the highest COVID-19 infection rate yet the lowest vaccination rate compared to the general population. The present study aimed to explore the rate of vaccine uptake as well as reported reasons for vaccine avoidance. In addition, we examined whether several protection motivation theory (PMT) components are good predictors of vaccine uptake. The components we addressed were: perceived susceptibility to the threat of COVID-19, perceived severity of the virus, and perceived efficiency and safety of the vaccine (i.e., response efficacy). The sample included 623 individuals (337 men) aged 18 + who were drawn from a database of a survey company specializing in the ultra-Orthodox community. We conducted a cross-sectional online survey between June 22, 2021, and July 7, 2021, approximately six months after the beginning of vaccination distribution. Results revealed that 65.8% of the participants (versus 89% of the general population) were vaccinated. Women were vaccinated at lower rates than men, whereas those in the Misnagdim ultra-Orthodox subgroup were vaccinated at higher rates than other subgroups in that community. The most prominent reasons for vaccine avoidance were perceived immunity based on prior infection by the virus and lack of trust in the vaccine’s safety. In support of the PMT model, the perceived severity of the virus and the vaccine high efficacy were significant predictors of vaccine uptake. The study results call for better outreach to this community and specific psycho-education interventions tailored for its women.

## Introduction

The COVID-19 pandemic caused an enormous number of victims and was considered a global crisis (Hiscott et al., 2020). By June 2021, the time of data collection for the present study, more than 180 million people globally had tested positive for the virus, and almost 4 million people had died. By that time in Israel, almost 850,000 had tested positive for the virus and 6,429 had died (Garti, 2022; JHU, 2023). Many countries enforced social distancing and lockdown measures, mask mandates, work-from-home orders, school closures, and restrictions on national and international travel to avoid spreading the virus (Öner et al., 2022). Simultaneously, tremendous resources were invested in finding a vaccine against the virus.

About a year after the outbreak of the pandemic, promising vaccines were created by the Pfizer, Moderna, and Johnson pharmaceutical companies, and they received Emergency Use Authorization (EUA) from the U.S. Food and Drug Administration (FDA) for persons aged ≥ 16 years (Self et al., 2021). The Pfizer vaccine imported to Israel by the Israeli Ministry of Health was 95% effective in preventing severe illness from the virus when two doses were provided (Dagan et al., 2021; Polack et al., 2020). It was also proven safe, with negligible adverse effects (Thomas et al., 2021). Nevertheless, the vaccination rate was far from expectation in many countries. For example, by the end of May 2021, around 40% of the U.S. population was vaccinated, and the vaccination rates varied by ethnicity, with lower rates among minority/racial groups (Brown et al., 2021). The vaccination rate in Israel by the end of February 2021 (at least one dose) was 53%. This rate was much lower among the two major minority groups in Israel—the Arab and ultra-Orthodox communities, despite having much higher COVID-19 infection and severe illness rates (Muhsen et al., 2021). By the end of June 2021, 89% of the Jewish population (ultra-Orthodox Jews excluded) were vaccinated with at least one dose, while only 59% of the ultra-Orthodox Jews were vaccinated (Knesset, 2021). The vaccination rate among the Jewish population (ultra-Orthodox Jews excluded) was somewhat lower in ages 40–49 (78.3%) and 30–39 (71.6%; Muhsen et al., 2021). This study addressed predictors of vaccination and the reported reasons for vaccination avoidance among the ultra-Orthodox Jewish community living in Israel.

## Literature Review

Israel ‘s ultra-Orthodox population (also known as the *Haredi* community) accounts for 12% of the country ‘s population (Malach & Cahaner, 2020). They live strictly according to Jewish law and tradition and separately from the majority (Tchernichovsky & Sharoni, 2015). Families are larger than among Israel’s other population sectors, and the living conditions are generally more crowded (Malach & Cahaner, 2020; Schnall, 2006). The ultra-Orthodox community is divided into three main subgroups: Hasidic, Lithuanian/Misnagdim, and Sephardim. Each subgroup has its unique characteristics, and they differ in lifestyles and worldviews (Gal, 2014). For example, the Hasidic gather around the court of the Rebbe—the movement ‘s spiritual leader who holds the community together. They centered on mysticism and devotion of the heart but divided by fidelity to their specific rabbinic leader. Misnagdim focused more rationally on cognitive powers and Torah study. They are considered a leading Torah group in the ultra-Orthodox community and therefore have the authority to set the behavior norms in all. Their devotion to Jewish law and tradition comes from their cognition rather than their heart. The Sephardim group includes Jewish descendants from Spain and the Islamic countries and other areas of Sephardic settlement. This subgroup gave rise to the SHAS political movement established in 1984, and as a result its power intensified in Israeli politics. At the same time, the Sephardic subgroup remained rejected by other subgroups of the ultra-Orthodox society and therefore engaged in building their own cultural and educational institutions. (Flint et al., 2012). Despite the differences, all the ultra-Orthodox subgroups are known for their devout observance of Jewish law and traditional values. Among these are learning the Torah as a significant value for adult ultra-Orthodox men and establishing families blessed with children (Braun-Lewensohn & Kalagy, 2019).

### The Ultra-Orthodox Community and COVID-19

During the COVID-19 pandemic, the ultra-Orthodox Jewish community in Israel was more vulnerable than the general population to the problems caused by COVID-19, with a much higher infection rate and rate of severe illness. The greater vulnerability was explained by crowded households, communal lifestyle, and limited exposure to external information on protective measures (Zalcberg & Block, 2021). In addition, the majority believed that the holy activity of Torah learning would protect them against the virus and that the protective measures imposed by the government contradicted their faith in almighty protection (Adini et al., 2022). Thus, even though the cumulative rate of infection among ultra-Orthodox communities was 2.5 times higher than in non-ultra-Orthodox ones (Weinreb, 2021), the odds of vaccine uptake among the ultra-Orthodox community were lower by 19% than the odds among the general Jewish population (Muhsen et al., 2021). Several theoretical models have aimed to explain individuals' motivation and barriers to health-protective behaviors in general and vaccination hesitance in particular. Among the leading ones is protection motivation theory (PMT).

### Protection Motivation Theory (PMT)

This theory argues that the individual undergoes a two-stage process before deciding to be engaged in a health-protective behavior: threat appraisal and coping appraisal. Within threat appraisal, the precursors of an intention to engage in protective behavior are threat susceptibility, threat severity, and maladaptive response rewards. Within coping appraisal, the determinants of an intention to adopt a given protective behavior are response efficacy, self-efficacy, and response costs (Floyd et al., 2000; Rogers & Prentice-Dunn, 1997). Thus, the likelihood that a person will engage in protective behavior is high when he or she assesses high susceptibility to the threat, high threat severity, that he or she is well able to perform the protective behavior, and that the protective behavior is highly effective and safe. In addition, the person should be convinced that both the cost of a protective response and the reward for a maladaptive response are low (Kothe et al., 2019). According to the PMT, in the case of the COVID-19 vaccine, the likelihood of someone getting the vaccine increases with high susceptibility; i.e., when a person is highly exposed to the virus directly (by getting the virus) or indirectly (having family members or friends who were sick or died); when the threat is perceived as severe (i.e., fears of COVID-19 effects are high); when the person perceives him/herself as fully capable of getting the vaccine; when the belief in the efficacy of the vaccine is firm, while the risk of short- and long-term vaccine side effects is low; and when there is no indirect benefit in getting sick from the virus. In this study, we explored the prediction of three components of PMT to the protective behavior of vaccine uptake: threat susceptibility, threat severity, and response efficacy. While the PMT model includes more components, it is accepted to rely on a few relevant components of the model in general (Floyd et al., 2000) and in the context of COVID-19 in particular (Acar & Kıcali, 2022; Hedayati et al., 2023; Kowalski & Black, 2021; Rahi, 2023).

### Supporting Evidence for the PMT

The fitness of the PMT model has already been studied with reference to COVID-19 protective measures such as social distancing and mask-wearing. For example, a May 2020 study conducted in Japan among almost 2,000 participants revealed that when the COVID-19 threat was perceived as severe and self-efficacy for adhering to the mitigation guidelines was high, adherence to COVID-19 protective measures was high (Okuhara et al., 2020). Another study conducted among almost 800 healthcare workers in Iran in March 2020 found that the associations between threat susceptibility, threat severity, self-efficacy, the efficacy of adherence to protective measures, and the low costs of this adherence were significant predictors of intention to adherence and actual adherence to COVID-19 protective measures (such as mask- and glove-wearing) (Bashirian et al., 2020).

As for the intention to vaccinate against COVID-19 and actually vaccine uptake, researchers have found mixed results. Whereas a few research studies found support for all PMT components in predicting intention and actual vaccination, others found support for only a few. For example, a study conducted in mainland China in June 2020 among more than 2,300 participants found that threat susceptibility, threat severity, self-efficacy, and internal rewards for maladaptive behavior (i.e., not getting the vaccine) were not significantly associated with the intention to get the COVID-19 vaccine, but the efficacy of the response and its costs were significantly associated with that intention (Li et al., 2021). In contrast, another study conducted among more than 3,000 students in higher education in mainland China found that threat severity was significantly associated with the intention of getting the vaccine, while the efficacy of the response, self-efficacy, and response costs were not associated with the intention to vaccine uptake (Wang et al., 2021). However, a study conducted in Iran among 265 participants that examined several components of the PMT—i.e., perceived susceptibility to COVID-19, perceived severity of COVID-19, perceived self-efficacy in performing the COVID-19 Vaccination, and perceived response efficacy of the COVID-19 vaccine—found that only perceived susceptibility to COVID-19 was not a significant predictor of vaccination intention, whereas all other components were significant predictors (Ansari-Moghaddam et al., 2021). Similarly, a study conducted among about 450 adults aged 50–64 in the U.K. found that all but one PMT component significantly predicted the intention to vaccine uptake; notably, perceived severity of COVID-19 was not associated with vaccination intention (Griffin et al., 2022).

In Israel, a study conducted among 309 participants in the first wave of COVID-19 and 240 in the second wave found that perceived susceptibility to COVID-19 and perceived severity of the virus were significant predictors of both intention and actual vaccine uptake. Lower associations were found between the perceived efficacy of the vaccine and both the intention to get vaccinated and vaccine uptake (Shiloh et al., 2022b). However, being an ultra-Orthodox Jew was negatively associated with vaccination (Shiloh et al., 2022a). Given the mixed support for the PMT model in predicting vaccination and the unique characteristics of the ultra-Orthodox community, we set research questions instead of hypotheses.

### Research Questions

1. What is the vaccination rate among ultra-Orthodox Jews in Israel?
2. What were the reported reasons for vaccine avoidance?
3. Will perceived susceptibility to the threat of COVID-19, perceived severity of the virus, and perceived efficacy and safety of the vaccine (i.e., response efficacy) predict the likelihood of being vaccinated?

## Methods

### Participants

The sample included 623 participants (338 men and 285 women) of 730 that were approached (85.3% response rate), aged 18 + who defined themselves as ultra-Orthodox Jews. Their average age was 32.19 (SD = 9.71), Median 30.0. Their median number of children was 3. About 80% of the participants (81.1%) were married. As far as subgroups within ultra-Orthodox society, about one-third (34.8%) defined themselves as Hasidic subgroup, 38.0% Lithuanian/Misnagdim subgroup, and more than a quarter (27.2%) as Sephardic. The sample was drawn from a growing database of 10,000 ultra-Orthodox people run and stored by the "Askaria" survey research company specializing in the ultra-Orthodox community in Israel. This survey institute has a team of researchers with direct knowledge of the ultra-Orthodox Jewish community. This knowledge enables them to face the challenges of adapting data collection and research tools to the ultra-Orthodox Jewish lifestyle and their cultural, social, and faith-based sensitivities. The database utilized in the present study included only those with Internet access or smartphones. According to publications of the Israel Democracy Institute (Malach & Cahaner, 2020), about two-thirds of the Israeli ultra-Orthodox community today have access to the Internet or a smartphone. Thus, the current sample does not represent the one-third of the ultra-Orthodox community that refuses to use the Internet or smartphones for religious reasons (Cahner, 2020). A stratified sampling of the various subgroups of the ultra-Orthodox community was prepared from the database stored in Askaria.

### Research Design and Procedure

The study is based on a cross-sectional research design with aspects (vaccine uptake) asked retrospectively. Following the approval of the Hebrew University, School of Social Work and Social Welfare ethics committee in June 16 2021, an anonymous online research questionnaire (using the Survey Monkey platform) was distributed by the Askaria survey research company. Informed consent was presented on the first screen, and only those marking "agree" were referred to the subsequent screens that introduced the research questionnaire. Data collection was held between June 22 and July 7, 2021, during the fourth wave of the pandemic in Israel, also known as the Delta variant wave, about six months after the beginning of the vaccine operation.

### Measurements

Independent variables included perceived susceptibility, perceived severity of the virus, and perceived response efficacy of the vaccine. The measurement scales for these variables were prepared for this study. One of the strengths of the PMT is its flexibility and adaptability to different phenomena and cultures (Tasantab et al., 2022); however, this strength has its costs. The PMT does not have a unified set of standard validated measurement scales. Most studies examining PMT efficacy in predicting health and other protective behaviors construct bespoke scales for each study (Kothe et al., 2019). Nonetheless, the variability in the measurement scales does not serve as a limitation to compare results between studies as a recent meta-analysis on the application of the PMT in preventive behaviors against COVID-19 suggests (Hedayati et al., 2023).

*Perceived susceptibility* was measured by three questions on the scope of COVID-19 infection: (a) "Has anyone in your family or close friends been diagnosed with COVID-19?"; (b) "Has anyone in your family been life-threateningly sick with the virus?"; and (c) "Has anyone in your family died from COVID-19?". Responses were provided on a 3-point scale: no one, one, more than one.

*The perceived severity of the virus* was measured by five statements referring to a different source of fear. Each statement had the opening of: "How much are you concerned about the following": "The number of people from the ultra-Orthodox community who have been infected with COVID-19"; "The number of people from the ultra-Orthodox community who have died from COVID"; "The fact that the virus is still spreading in different parts of the world"; "The governmental restrictions that have been imposed to mitigate the spreading of the virus"; and "The economic crisis and its consequences. The response scale ranged from 1, "not concerned about it at all," to 5, "very much concerned about it." Inter-item reliability was adequate (α Cronbach = 0.73).

*Response efficacy* was measured by trust in the efficiency: "I’m not sure the vaccine is efficient against all the virus variants" and safety of the vaccine: "It is unclear whether the vaccine may cause harm in the long run". The response scale ranged from 1 "not concerned about it at all" to 5 "very much concerned about it." Inter-item reliability was adequate (α Cronbach = 0.75).

*Vaccine uptake* (the dependent variable) was measured by one question: "Have you been vaccinated against the COVID-19 virus?" The answers were: “yes, in two doses," "yes, in one dose," and "no." The two "yes" categories collapsed, forming dichotomous vaccination categories: yes/no.

*Perceived reasons for vaccine avoidance-* those who reported "no" on the vaccine uptake question were asked about 15 potential reasons for not getting the vaccine (e.g., “I do not trust the drug companies that the vaccine is safe") and an "other" category. The items are presented in Table 2. Several items were driven from other studies (Gewirtz-Meydan et al., 2022), while the rest were prepared specifically for the present study.

*Background* variables of gender, age, number of children, and religious subgroup were based on one self-report question for each variable.

### Data Analyses

Analyses were conducted using SPSS 27. Descriptive statistics on vaccine uptake and reasons for vaccine avoidance were followed by a 2-group (vaccinated and not vaccinated) comparison of all study variables. We then performed two hierarchical logistic regression analyses, one with background variables and one with all study variables.

## Results

### COVID-19 Experiences and Attitudes Toward the Vaccine

About two-thirds (65.8%) of the participants were vaccinated. Table 1 presents COVID-19 experiences and attitudes toward the vaccine among those vaccinated and those who were not. It shows that exposure to COVID-19 experiences (susceptibility) was significantly higher among those who *were not* vaccinated (t(471.936) = 6.348, p < 0.001). The perceived reasons for vaccine avoidance presented in Table 2 may explain this unexpected finding. It shows that 63.2% of the respondents who were not vaccinated reported their reason as "I recovered from COVID-19, and I am immune." Thus, exposure to COVID-19 experiences may be perceived as less vulnerability rather than greater. As expected, the level of response efficacy, i.e., perceived efficiency and safety of the vaccine, was higher among those who were vaccinated (t(369.872) = 6.468, p < 0.001).

**Table 1:** Participant demographic characteristics and description of the study variables in the total sample and by vaccine uptake

	Total sample	Vaccinated	Not Vaccinated
	N = 623	N = 410 (65.8%)	N = 213 (34.2%)
	Mean(SD)	Mean(SD)	Mean(SD)
Background and research study variables			
Age	32.19 (9.71)	32.75 (10.02)*	31.11 (9.00)
Number of children	3.83 (2.27)	3.87 (2.32)	3.76 (2.19)
Threat susceptibility (level of exposure to COVID-19)^1^	5.24 (1.59)	4.97 (1.60)	5.77 (1.44)***
Threat severity (fears of COVID-19)^2^	2.56 (0.86)	2.59 (0.86)	2.49 (0.84)
Response efficacy (level of perceived efficiency and safety of the vaccine)^3^	3.56 (1.15)	3.79 (1.04)	3.14 (1.24)***

	%	%	%
Gender			
Men	54.2	69.4	30.6
Women	45.8	61.4	38.6*
Subgroups of ultra-Orthodox society			
Hasidic	34.8	61.9	38.1
Misnagdim	38.0	74.7***	25.3
Sephardic	27.2	56.7	43.3
Self-rated health			
Very good	70.0	64.7	35.3
Good	27.1	68.5	31.5
Not so good/bad ^4^	2.9	–	–

^1^On a scale from 1 to 10. Higher score indicates more exposure

^2^On a scale from 1 to 5. Higher score indicates greater fears

^3^On a scale from 1 to 5. Higher score indicates greater perceived efficacy of the vaccine

^4^This category was removed from the comparison between vaccinated and not vaccinated due to the small number of cases

*p < .05 **p < .01 ***p < .001

**Table 2:** Reported reasons for vaccine avoidance in descending order

Reason	Total sample (228)	Men (n = 110)	Women (n = 118)	Hasidic or Sephardic (n = 154)	Misnagdim (n = 61)
	%	%	%	%	%
I recovered from COVID-19 and I am immune	63.2	65.5	61.0	61.0	70.5
I still do not know enough about the harm that the vaccine may cause in the long run	36.0	25.5	45.8**	39.6	26.2
COVID-19 is not dangerous for people of my age	20.2	24.5	16.1	22.7	11.5
I am afraid that the vaccine will harm my fertility	18.9	10.0	27.1***	22.7**	6.6
I do not trust the drug companies that the vaccine is safe	16.2	11.8	20.3	20.8*	6.6
I do not believe that the vaccine is effective	15.4	10.0	20.3*	18.8**	3.3
I am exercising my legal right not to be vaccinated	15.4	48.2	51.8	17.5	8.2
I’m pregnant/trying to get pregnant/nursing	13.6	0.0	26.3***	14.9	9.8
I do not trust the government to make sure that the vaccine is safe	13.2	8.2	17.8*	16.9**	3.3
I do not believe in the safety of the vaccine in the short term	7.9	5.5	10.2	9.1	3.3
I prefer not to put drugs or chemicals in my body	6.6	5.5	7.6	7.1	4.9
I do not believe I should get vaccinated if others get vaccinated	5.3	7.3	3.4	6.5	3.3
My rabbi told me not to get vaccinated	5.3	6.4	4.2	4.5	8.2
It's hard for me to get to the vaccination site	4.4	4.5	4.2	5.2	3.3
It is not customary in our community to get vaccinated	2.6	4.5	0.8	3.2	1.6
Other (I’m not in a rush to get vaccinated; if the vaccine so effective why do we need several vaccine doses; I’m afraid of needles)	11.0	18.2***	4.2	7.1*	18.0

As for differences in vaccination by background variables, women reported a lower rate of vaccination (χ^2^(1) = 4.425, *p* = 0.035). This finding may be partially explained by fear of the vaccine harming their fertility or because they were pregnant or nursing and did not want to risk the fetus/baby, as shown in Table 2. The rate of vaccination among those who identified as Misnagdim subgroup was higher than participants who identified with other subgroups (χ^2^(1) = 14.199, *p* < 0.001).

### Reasons for Vaccine Avoidance

The most frequently reported reasons for vaccine avoidance were (a) recovery from COVID and, therefore, a sense of immunization and less susceptibility (63.2% of the respondents indicated this reason); (b) disbelief in the vaccine effects in the long run (36.0%); and (c) perceived low vulnerability to the virus in younger ages (20.2%). Women were more skeptical about the effectiveness of the vaccine (χ^2^ (1) = 8.565, p = 0.003) and had less trust in the government to assure its safety (χ^2^(1) = 4.606, p = 0.032). Women were also more concerned about the harm the vaccine might cause in the long term (χ^2^(1) = 10.195, p = . 001) and that it might harm their fertility (χ^2^(1) = 10.092, p < 0.001).

As for differences among the subgroups, the Hasidic and Sephardic subgroups were more skeptical than Misnagdim about the effectiveness of the vaccine (χ^2^(1) = 8.565, p = 0.003); had less trust in the drug companies (χ^2^(1) = 6.339, p = 0.012) or the government (χ^2^(1) = 7.139, p = 0.008) to assure their safety; and were more concerned about potential harm the vaccine might cause to their fertility (χ^2^(1) = 7.694, p = 0.006).

### Prediction of Vaccination

Table 3 presents hierarchical logistic regression for vaccination. The logistic rather than linear regression was used because the predicted variable, vaccination, was binary (at least one dose, none). Similar to the bivariate analyses (presented in Table 1), exposure to COVID-19 experiences was associated with *less* vaccination after controlling for background variables; exposure to people that were infected, sick, and had even died of COVID-19 *reduced* the odds of vaccine uptake by 27.6% (OR = 0.724 CI = 0.626–0.837, *p* < 0.001). In accordance with our expectation, those holding high threat severity of COVID-19 (fears of COVID-19) are twice as likely to be vaccinated as those who hold low threat severity (OR = 2.172 CI = 1.583–2.979, *p* < 0.001). Similarly, those who have a greater belief in the efficacy of the vaccine (high response efficacy) are twice as likely to be vaccinated as those with less belief in the efficacy of the vaccine (OR = 2.148 CI = 1.694–2.723, *p* < 0.001).

**Table 3:** Hierarchical logistic regression predicting getting vaccination

Variables	Model 1		Model 2	
	Odds ratio (95% CI)	*P* value	Odds ratio (95% CI)	*P* value
Gender (women)	.730 (.485, 1.099)	.132	1.025 (.641, 1.638)	.918
Age	1.016 (.987, 1.045)	.294	1.015 (.983, 1.048)	.357
Number of children	1.007 (.899, 1.128)	.905	.997 (.879, 1.131)	.963
Lifestyle				
Hasidic ^1^	1.079 (.660, 1.764)	.763	1.030 (.600, 1.769)	.915
Misnagdim^1^	**2.608 (1.546, 4.401)**	** < .001**	2.274 (1.283, 4.032)	** < .01**
Nagelkerke R Square	.068	< .001		
Threat susceptibility—level of exposure to COVID-19			**.724 (.626, .837)**	** < .001**
Threat severity—fears of COVID-19			2.172 (1.583, 2.979)	** < .001**
Response efficacy- level of perceived efficiency and safety of the vaccine			2.148 (1.694, 2.723)	** < .001**
Nagelkerke R^2^			.263	< .001

^1^Subgroups were entered as dummy variables. The referent group was Sephardic

Bold values reflect the significant predictors

Nagelkerke R Square measures the goodness of fit in logistic regression analysis. It ranges from 0 to 1, with values closer to 1 indicating a better fit of the model. Where R Square in linear regression can be interpreted as the proportion of variance explained by the model, Nagelkerke R Square cannot be interpreted as such

## Discussion

This study addressed the vaccination rate, reported reasons for vaccine avoidance, and motives for vaccination based on the protection motivation theory (PMT). Results revealed that two-thirds of the participants were vaccinated with at least one dose. This rate was somewhat lower than among the general Jewish population when compared to published data (Muhsen et al., 2021). We nevertheless found differences in vaccine uptake within the ultra-Orthodox community. The vaccination uptake rate among women was significantly lower than that of men. This result is supported by previous studies conducted in other countries among non-Jewish population (Gaffney et al., 2022; Zintel et al., 2022) and Israel (Shiloh et al., 2022b). While a few of the reported reasons for vaccine avoidance among women, such as lower (than men's) belief in its safety, are universal (Alleaume et al., 2021; Bono et al., 2021), others, such as being pregnant or high concern for its potential harm to fertility, were more prominent among the ultra-Orthodox community in Israel. Specifically, traditional Jewish legal sources emphasize the divine command (mitzvah) to bring children into the world. “Each man must marry a wife to be fertile and multiply” (*Shulchan Arukh, Even HaEzer 1,1*). Also, the study participants' average age (32) meant they were still in their high fertility life cycle stage. A study conducted at the Haredi Institute for Public Affairs found a significant gap in the readiness for vaccine uptake between young ultra-Orthodox women (ages 18–44) and older ones (ages 45 +). In the group of younger women, 72.1% reported refusal to be vaccinated against COVID-19, while 51% of older women reported vaccine refusal. No age differences were found among men (Krombi & Berenbloom, 2021). Thus, the risk of harming fertility may be perceived by ultra-Orthodox women as a potential violation of a major religious role and the core role of women in this community. Future studies should further examine this potential explanation.

### Differences by Sub-Groups Among the ultra-Orthodox Community

The Lithuanian/Misnagdim subgroup had a higher rate of vaccination uptake and lower mistrust of its efficacy among those who were not vaccinated compared with the Hasidic and Sephardic ultra-Orthodox subgroups. We can interpret this finding by the characteristics of the Misnagdim. They are oriented toward analytical thinking, and many are open to more secular information, such as scientific data and publications (Braun-Lewensohn & Kalagy, 2019). In fact, during COVID-19, the majority of the ultra-Orthodox journalists who used the Twitter platform to spread information on the risk of COVID-19 belonged to the Misnagdim stream (Shomron, 2022). Thus, this group’s greater exposure to information and data from doctors and scientists who repeatedly emphasized the efficiency and safety of the vaccine probably persuaded some of them to get vaccinated.

### Present Findings and the PMT Model

The present findings provide partial support for the PMT theoretical model. In accord with PMT (Rogers & Prentice-Dunn, 1997) and previous research studies (Griffin et al., 2022; Yahaghi et al., 2021), the odds of vaccine uptake were much higher when threat severity of COVID-19 and belief in vaccine response efficacy (i.e., efficiency and safety of the vaccine) were high. Nonetheless, higher susceptibility to COVID-19 was not associated with higher vaccine uptake. In fact, higher susceptibility was associated with *lower* vaccine uptake. This unexpected finding may have several explanations. First, high exposure to the virus may create an illusion of being immune, leading to downplaying the risk of COVID-19 based on personal or family’s/friends’ experiences and therefore the perception of less necessity for the vaccine (Qiao et al., 2021). Supportive evidence for this interpretation can be drawn from a study conducted in Italy among 600 participants who had experienced acute COVID-19 with mild illness that needed inpatient or outpatient hospitalization care. That study revealed that almost 60% were hesitant or undecided about COVID-19 vaccine uptake (Gerussi et al., 2021). Indeed, findings from our study on the perceived reasons for vaccine avoidance also support the perceived immunity interpretation. The most frequent reason for vaccine avoidance (63%) was "I recovered from COVID-19 and am immune." Thus, susceptibility to a health threat may be a two-edged sword. On the one hand, it may increase motivation for health-protective behavior. On the other hand, it may serve as a barrier to taking steps toward health-protective behavior. Perhaps another component in the PMT theory (i.e, the response cost of executing the protective behavior - vaccine uptake), not addressed directly in this study, may explain which road the person takes (Prentice-Dunn & Rogers, 1986). The findings in the present study suggest that the ultra-Orthodox individuals who were not vaccinated were concerned with the health cost of the vaccine in the long term and, if addressed in the prediction model, might have served as a moderator between susceptibility to COVID-19 and vaccine uptake. Nonetheless, one cannot rule out the possibility that the high susceptibility to COVID-19 due to direct infection by the virus may have served as at least partially objective immunization as several countries did declare that COVID-19 infection is immune the person from regaining the virus for three to six months ahead.

Interestingly and similarly to Jacobson et al. findings (Jacobson et al., 2023), barriers to vaccination among the ultra-Orthodox Jews are not religious-framed but more related to lack of knowledge, fears, trust, and logistics. Perhaps one exception is the high rates of fear of harm the vaccine may cause to fertility, especially among women, which was indicated as a barrier to vaccination. The significant barrier to the fear of harming fertility may be related to the high values the ultra-Orthodox society holds for large families, child-rearing, and transmitting their religious values to the next generation (Pirutinsky et al., 2015).

## Limitations

Study limitations include its cross-sectional, partially retrospective design, the tailor-made measurement scales, and the fact that not all PMT components were addressed. In addition, this study excluded one-third of the ultra-Orthodox community that refuses to use the Internet or smartphones for religious reasons. Due to budget constraints, we were reluctant to integrate other data collection methods (e.g., face-to-face interviews). Finally, while the statistics among the Ultra-Orthodox Jews indicated that 27% of the adult population in this society are ages 45 + , our sample included only 10.5% of 45 + . Thus, this study does not represent well the conservative ultra-Orthodox Jews and those who are ages 45 and above. A longitudinal study using face-to-face data collection methods, a better representation of older ages and the conservative ultra-Orthodox Jews, with all PMT components on vaccine uptake, especially the physical and emotional potential costs of the vaccine and other protective behavior among the ultra-Orthodox community, is recommended. We also need further understanding of women's fears of fertility harm if vaccinated and the critical role it plays in the lives of ultra-Orthodox women as well as mechanisms for mitigating their fears.

## Implications

Given the fact that the vaccination rate among the ultra-Orthodox Jewish community is lower than the one in the general population on various types of vaccines (Jacobson et al., 2023; Muhsen et al., 2012), the implication to practice includes providing extensive information through the media channels explicitly targeted to this community to address the significance of vaccination. In addition, the information and psycho-educational interventions provided by professionals and rabbis should be differentiated by gender and subgroups within ultra-Orthodox society.
